# Multidisciplinary Treatment in Toxic Epidermal Necrolysis

**DOI:** 10.3390/ijerph20032217

**Published:** 2023-01-26

**Authors:** Agnieszka Surowiecka, Wioletta Barańska-Rybak, Jerzy Strużyna

**Affiliations:** 1 East Center of Burns Treatment and Reconstructive Surgery, Medical University of Lublin, 20-059 Lublin, Poland; 2 Department of Dermatology, Venereology and Allergology, Faculty of Medicine, Medical University of Gdańsk, 80-210 Gdańsk, Poland; 3 Department of Plastic Surgery, Reconstructive Surgery and Burn Treatment, Medical University of Lublin, 20-059 Lublin, Poland

**Keywords:** toxic epidermal necrolysis, IVIGs, plasmapheresis, dressings

## Abstract

Toxic epidermal necrolysis, Leyll’s syndrome (TEN), is a rare mucocutaneous blistering disease burdened with high mortality rates. The diagnosis of TEN is based on clinical symptoms and histopathological findings. In approximately 90% of cases, it is a severe adverse reaction to drugs. In TEN, not only is the skin affected, but also mucosa and organs’ epithelium. There are no unequivocal recommendations in regard to systemic and topical treatment of the patients. The aim of this paper is to review available literature and propose unified protocols to be discussed. Early management and multidisciplinary treatment are necessary to improve patients’ outcome. Treatment of patients with TEN suspicions should be initiated with early drug withdrawal. TEN patients, like patients with burns, require intensive care and multidisciplinary management. Each patient with TEN should be provided with adequate fluid resuscitation, respiratory support, nutritional treatment, pain control, infection prophylaxis, anticoagulant therapy, and gastric ulcer prophylaxis. The key to local treatment of patients with TEN is the use of nonadherent dressings that do not damage the epidermis during the change. The aim of the systemic treatment is purification of the blood stream from the causative agent. The most efficient way to clarify serum of TEN patients’ is the combination of plasmapheresis and IVIG. Immunomodulatory therapy can reduce the mortality five times in comparison with the patients with immunosuppression or lack of full protocol.

## 1. Introduction

Toxic epidermal necrolysis, Leyll’s syndrome (TEN), is a rare mucocutaneous blistering disease [1,2,3], with a frequency of 0.4–1.9 cases per million per year [4,5]. In approximately 90% of cases, it is a severe adverse reaction to drugs (ADR) [6]. Severe cutaneous adverse reactions (SCAR) have a high mortality rate, and their exact pathogenesis is still not fully understood [1]. The onset of TEN may also be associated with infectious agents such as Mycoplasma pneumoniae, cytomegalovirus, varicella zoster virus, Epstein–Barr virus, and HIV [2]. There is also a case of simultaneous manifestation of TEN and SARS-CoV-2 infection described in the literature [7].

The diagnosis of TEN is based on clinical symptoms and histopathological findings [4,8]. Prodromal syndromes, such as fever or flu-like symptoms, precede the appearance of erythema, blisters on the skin with Nikolsky’s symptom, and erosions on the mucosa [4]. Toxic epidermal necrolysis differs from Stevens–Johnson syndrome (SJS) in terms of a greater extent of lesions. In TEN, more than 30% of the total body surface area (TBSA) is affected, and the recorded mortality is higher. According to the available data, the mean mortality among patients with TEN treated in the ICU is currently around 30–60% [2,9,10,11]. The recently observed decrease in mortality may result from better wound care and sepsis control [12]. There are many factors that increase mortality in TEN. Leukopenia, anemia, hyperglycemia [2], and coagulopathies [13,14] are known mortality risk factors. Patients diagnosed with TEN require multidirectional and multidisciplinary treatment. In most cases, they are hospitalized in intensive care units (ICU) or burn units and require multidirectional prevention of organ complications. One of the most serious complications in patients with TEN is multiorgan failure (MOF).

Lesions observed in TEN are caused by infiltration of the epidermis by cytotoxic T lymphocytes and NK cells without destruction of the basal layer [1,4]. The second pathway leading to epidermis necrolysis promotes apoptosis of epithelial cells by activation of the Fas-FasL ligand. The third potential mechanism is lymphocytes’ secretion of granulysin and the perforin–granzyme complex. Multiorgan lesions are secondary to pro-inflammatory cytokines and death mediators circulating in the blood stream and infectious complications [1,4,6]. Elevated levels of tumor necrosis factor (TNF), interferon γ(INF-γ), IL-6, IL-1, IL-10, and IL-18 can be detected in patients’ serum or blisters’ fluid [1,6]. IL-17 enhances the action of INF-γ and stimulates keratinocytes to additionally secrete pro-inflammatory cytokines [15]. Serum IL-15 and granulysin levels are associated with higher mortality and higher SCORTEN [16,17]. In approximately 97% of cases, skin lesions are accompanied by erosive lesions of the mucosa [18].

## 2. Diagnosis

A prompt diagnosis of TEN is crucial for discontinuing the aggravating substance and initiating supportive and complementary therapy to improve health outcomes. Nonetheless, the clinical presentation can be comparable to a variety of other blistering diseases, and diagnosis is not always clear. TEN is a severe dermatosis connected with epidermal loss and mucositis on several sites, as well as systemic symptoms. Toxic epidermal necrolysis can be differentiated as overlap SJS–TEN—detachment or skin necrosis of 10–30% BSA plus widespread purpuric macules or flat atypical targets—or TEN—detachment or skin necrosis greater than 30% BSA. A distinctive feature is preceding mucocutaneous lesions by prodrome: fever, malaise, sore throat, and upper respiratory tract symptoms, i.e., cough, dyspnea, bronchial hypersecretion, hemoptysis by several days. Cutaneous pain is a common early sign of TEN, which presence should alert the clinician to the onset of epidermal necrolysis. Atypical lesions and/or purpuric macules are the first lesions to appear. The upper torso, proximal limbs, face, and mucosal sites (eyes, mouth, nose, genitalia) are frequently the first areas to be affected. Following that, the lesions coalesce and spread to the rest of the trunk and distal limbs, reaching a maximum of 5–7 days after disease onset, evaluating in terms of the blisters. The necrotic epidermis separates from the underlying dermis, causing erosion development. These lesions can result in bleeding and infection leading to systemic inflammation. Involvement of necrolysis in the respiratory tract epithelium, gastrointestinal epithelium leading to profuse diarrhea, and acute tubular necrosis leading to acute kidney injury may occur in the early phase of TEN, thus the multidisciplinary approach to the TEN patients is recommended [19].

Excluding the differential diagnosis of staphylococcal scalded skin syndrome (SSSS) by clinical examination of mucosae (which are not involved in SSSS) and skin biopsy if there is any diagnostic question is critical in making an appropriate diagnosis. Additionally, it is highly needed to find out all medicines taken and vaccinations received by patients over the preceding 2 months, including over the counter and complementary/alternative therapies, and discontinue any potential TEN-causing drug right away, Table 1.

It is recommended to conduct a validated prognostic scoring system, SCORTEN, in all patients with TEN within the first 24 h of admission. It allows predicting the probability of hospital mortality based on seven occurring parameters [19]. The diagnosis of TEN is clinicopathological as it relies largely on clinical findings. Differential diagnosis and disease confirmation are made based on clinical features with histopathological purposes [20].

## 3. Histopathology

Histologically, there is a wide range of epidermal damage, from individual cell death to confluent epidermal necrosis. Epidermal alterations relate to basal cell vacuolar degeneration and the production of subepidermal vesicles or bullae. Early lesions are characterized by sparse superficial perivascular and interstitial lymphocytic infiltrates, some lymphocytes at the dermo–epidermal junction, and necrotic keratinocytes scattered throughout the lower epidermis and, occasionally, the upper part of the infundibular epidermis and eccrine ducts. Furthermore, subepidermal vesiculation appears owing to widespread necrotic keratinocytes in fully established lesions, culminating in confluent epidermal necrosis. The basket-weave pattern is preserved in the cornified layer [21].

## 4. Differentiation

### 4.1. Staphylococcal Scalded Skin Syndrome (SSSS)

Staphylococcal scalded skin syndrome (SSSS) is a condition caused by epidermal detachment induced by exfoliative exotoxins produced by *Staphylococcus aureus*. Patients often present with fever, erythema, and painful skin, followed by blistering. Both syndromes’ bullae present a positive Nikolsky sign, however, SSSS demonstrates a Nikolsky sign on unaffected skin [22]. Clinically, SSSS is distinguished from TEN by the absence of mucous membrane involvement and superficial epidermal peeling, as opposed to the full thickness stripping seen in TEN. The presence of superficial blistering through the granular layer in biopsy tissues confirms the SSSS clinical impression [23].

### 4.2. Erythema Multiforme Major (EMM)

In contrast to the systemic involvement of TEN, erythema multiforme major is a localized eruption of the skin and mucous membranes. EMM lesions are target lesions that are positioned on the periphery of the skin and have minimal epidermal detachment. Despite many lesions, EMM generally involves less than 10% of the body surface area, predominantly localized on the limbs and extremities. Mucous membrane involvement is either lacking or restricted to a single area, most commonly the mouth [24]. EMM is mostly related to herpes simplex virus (HSV) reactivation and rarely to drugs, whereas TEN is typically triggered by medications, seldom by an infection [19].

### 4.3. Linear IgA Bullous Dermatosis (LABD)

Linear IgA bullous disease (LABD) is a relatively rare subepidermal vesiculobullous autoimmune disease that can occur in both adults and children [25]. The disease is subclassified into idiopathic and drug-induced forms [26]. It is defined by linear IgA deposition along the basement membrane zone and rupture of the dermo–epidermal junction. In adults, LABD appears as tense vesicles and bullae that combine to form annular or heterocyclic plaques, often with an erythematous base [27]. Lesions most commonly affect the trunk and extension body areas, nevertheless, they can occur on any part of the body. Although rare, the oral cavity and the eyes may also be involved. The detection of linear IgA deposits along the basement membrane zone in the immunopathological examination is the gold standard in the diagnosis of LABD. The presence of these deposits may persist for up to several months after the skin changes have disappeared.

### 4.4. Other Diseases

Other diseases that require differentiation from TEN include acute generalized exanthematous pustulosis (AGEP), autoimmune bullous diseases (e.g., bullous pemphigoid, paraneoplastic pemphigus), autoimmune diseases (e.g., bullous lupus erythematosus), and generalized fixed bullous drug eruption.

#### Early Management

Toxic epidermal necrolysis not only affects epidermal and mucosal membranes but also can cause complications in several organs, such as the liver, kidneys, and respiratory tract. Thus, there is a high need to conduct a multidisciplinary approach and early management for TEN patients. Some research on TEN has shown that prompt admission to a burns center is linked to a higher chance of survival and warrants improved patient outcomes [28,29]. In the early stages of TEN management, it is critical to discontinue taking any causative drugs. What is more, supportive treatment such as fluid replenishment [30], pain medication, nutritional evaluation [31], and supplementary oxygen are indicated.

According to current U.K. recommendations, to rule out an immunobullous disorder, a biopsy from lesional skin just adjacent to a blister should be sent for routine histopathology, and a second biopsy from periblister lesional skin should be sent unfixed for direct immunofluorescence. What is more, swabs from lesional skin ought to be directed for bacteriology [19].

Since the necrotic epidermis in TEN is prone to detach from the underlying dermis, it is critical to handle the skin with care in these patients. In accordance with the U.K. guidelines, specialist nurses who are experienced with skin fragility diseases should provide day-to-day bedside treatment. The crucial aspect is to inform other attending clinicians before examining the patient about the patient’s problems with an epidermal detachment to minimize possible skin damages during the examination [19].

There are several methods of treating lesional skin locally in these patients, however, there is no evidence, and therefore no guidelines, which would indicate the most proper one. Multiple techniques for local care of lesional skin are available, and there is no conclusive evidence as to which is best. The detached epidermis can be left in place to function as a biological dressing for the underlying dermis in the conservative approach. When there are substantial bullae, the blister fluid should be aspirated or released, and the blister roof should be allowed to settle over the underlying dermis. During the acute phase of TEN, it is beneficial to apply a bland, greasy emollient to the whole skin (including denuded areas) on a regular basis to limit transcutaneous water loss, improve barrier function, and induce re-epithelialization. It is important to avoid any products that include irritants. The application of an adequate, nonadherent dressing to the exposed dermis is crucial as it can limit microbial colonization, reduce fluid loss, and aid in pain control. What is more, covering the affected skin may help hasten the process of re-epithelialization. In TEN, the denuded dermis leaks serum and is covered with necrotic debris. Microbial colonization occurs on the exposed dermis, which is first colonized by *Staphylococcus aureus* and then by Gram-negative bacteria from the gut microflora, particularly *Pseudomonas aeruginosa* [32,33]. Cutaneous infection slows re-epithelialization and can progress to systemic sepsis, which is the leading cause of mortality in TEN patients. Nonetheless, antimicrobial therapy should only be started if there is clinical evidence of infection, as indiscriminate use of preventive systemic antibiotics might enhance skin colonization [34].

In case of failure of conservative types of management, a surgical approach can be considered [19]. Under general anesthesia, removal of the necrotic infected epidermis, cleaning wounds with a topical antibacterial treatment (e.g., betadine or chlorhexidine), and debridement of the detached epidermis to eliminate possibly infectious material followed by physiological wound healing with biosynthetic dressings, xenograft, or allograft are recommended, Table 2. These more aggressive procedures are usually performed in a burn center [35]. Most of the patients with confirmation of TEN should be considered to be transferred to burn units with available intensive care. The transfer should be planned, agreed between the units, and proceeded with after the stabilization of the patient’s condition. Helicopter Emergency Medical Service (HEMS) should be considered prior to ground transportation when the estimated time of transport to the referent department is more than an hour on wheels or the patient’s condition is severe [36,37]. Early referral to a burn unit should be considered; however, national procedures should be established to improve arrangements between dermatological and surgical units.

## 5. Symptomatic Treatment

Treatment of patients with TEN suspicions should be initiated with early drug discontinuation [1,2,18,38].

TEN patients, like patients with burns, require intensive care and multidisciplinary management. Both pathologies are manifested by skin damage and a systemic inflammatory reaction. Patients with TEN lose fluids and proteins as a result of the destruction of the epidermal layer [10]. Therefore, intensive fluid resuscitation should follow the recommendations for burn patients [2].

Additionally, the mechanisms leading to hypovolemia, acute early renal failure, and sepsis are similar to those of burns [39]. Therefore, supportive care for patients with extensive skin loss should follow the same guidelines as for severe burns. Each patient with TEN should be provided with adequate fluid resuscitation, respiratory support, nutritional treatment, pain control, infection prophylaxis [38], anticoagulant therapy, and gastric ulcer prophylaxis [40].

Table 3 summarizes key points in symptomatic treatment.

### Wound Control

Proper wound care provides protection against infection [12]. A total of 75% of the units disclosed the method of wound care, but as many as 80% of them reported the use of “any type” of dressings. Almost half of the centers, 46.6%, admitted using dressings with antibacterial properties [12,41,42]. Interestingly, less than 2/3 of the US burn centers admit that they have developed a procedure for treating patients with TEN. Castillo analyzed 13 available publications on the use of dressings in TNN. Most of the works are descriptions of individual cases; therefore, based on the data obtained, it is impossible to create reliable recommendations. The main advantage of biosynthetic dressings is the possibility of a single application, but they are associated with high costs and the risk of infection [43]. There are reports in the literature on the effective use of alloplastic dressings based on caprolactone, polyactide and trimethylene carbonate [44], or collagen matrices. Dressings ensured proper control of the wound environment and re-epithelialization for up to approximately 15 days.

Burn centers treating patients with TEN can be categorized as “aggressive” or “expectant”. The first type of units prefers early surgical debridement of wounds, primary usage of a water knife, and the use of biosynthetic dressings. The “waiting” centers postpone the decision of surgical intervention. The analysis of mortality and re-epithelialization time shows that the data are inconclusive and the end results are similar [19].

The key to local treatment of patients with TEN is the use of nonadherent dressings that do not damage the epidermis during the change [12]. The main goals of dressings in TEN are protection against conversion and/or infection, stimulation of epithelization, protection against fluid loss and hypothermia, and pain control [19]. It is recommended to manipulate the patient gently, minimize shear forces, and avoid actions that may damage the epidermis. Preparations containing sensitizing or irritating substances should be avoided. Oily emollients are used on unchanged skin, Table 4 [19].

Topical antibacterial agents should be reserved only for necrotic areas [19]. The most commonly used products are those containing silver, but one should bear in mind the risk of absorption of silver ions by large wound surfaces and the suppressive effect of silver on the epidermis. The mechanism of action of nanocristalic silver is complex. It generates reactive oxygen species, lipid peroxidation, bacterial membrane disintegration, and has a synergic effect with systematic antibiotics. However, there are some cases of bacterial resistance reported [45].

According to dermatological society guidelines, treatment of patients with toxic epidermal necrolysis has many similarities with burn care. However, they do not recommend early surgical interventions, but rather conservative treatment [46]. The group marked a need of constant inflammation prevalence in order to decrease mortality and improve outcomes [46].

Systematic treatment is essential in TEN. Difficulty in decision making of what type of immunomodulatory treatment to choose arises from a low number of patients, lack of multicenter observations, and differences in clinical experiences. Krajewski et al. performed a meta-analysis and meta-regression on the impact of medical interventions on mortality, length of hospital stay, and re-epithelialization time in TEN. A total number of 42 studies were included in the pooled synthesis. The lowest mortality rate was found to be associated with etanercept. The time to complete re-epithelialization was 13.278 days (95%CI, 8.773–17.784). The longest treatment time was found with cyclosporine treatment, 14,739, while the lowest was found with glucocorticoids [47]. Systemic corticosteroids in pulses of 1–2 mg/kg showed no effect on mortality in TEN [10]. Corticosteroids inhibit inflammatory cells and the release of cytokines. Two trials, EuroSCAR and RegiSCAR, showed contradictory results regarding the effect corticosteroids have on mortality [48,49]. Cyclosporin in an average dose of 3–10 mg/kg inhibits T cells, NK cells, and cytokine production [2]. The 2017 JAMA trial found several favorable outcomes of cyclosporin [37]. Furthermore, RegiSCAR revealed beneficial effects as well [16]. Guidelines on dosage and duration of cyclosporine administration in TEN are not available [48]. TNF-alpha inhibitors were first used in 2002 (infliximab in a single dose of 300 mg). Etanercept is an inhibitor of TNF and impedes cell apoptosis. A single dose of 50 mg subcutaneously was administrated [2]. Re-epithelization time after anti-TNF agents was shorter in comparison with corticosteroids [49]. What is more, anti-TNF agents reduce plasma and blister fluid granulysin levels [49]. There are several reports on a combination therapy consisting of anti-TNFs with corticosteroids and IVIG [50,51,52].

## 6. Conclusions

The most important action in TEN is to stop the delivery of the causative agent. Early recognition of the potential trigger is necessary to state the suspicion of SCAR. In TEN, not only is the skin affected, but also mucosa and organs’ epithelium. The knowledge regarding organ lesions is rather empirical. It manifests with bleeding from the gastrointestinal system or genitourinary tract. It can be assumed that organ changes are due to circulating immunological compounds or the residual drug and its metabolites. That is, early management and multidisciplinary treatment are necessary to improve patients’ outcomes.

## Figures and Tables

**Table 1:** Differentiation of TEN with other blistering disorders.

Symptoms	Time of Occurrence	Duration	Location	Treatment
Prodrome (i.e., fever, malaise, sore throat, and upper respiratory tract symptoms)	Days to weeks after drug application	Over a period of 1 day to 2–3 weeks	–	Symptomatic treatment
Atypical lesions and/or purpuric macules (confluent erythema in severe cases)	Several days (1–3) after prodrome	Several days (reaching a maximum of 5–7 days after disease onset)	Upper torso, proximal limbs, face, mucosal sites (i.e., eyes, mouth, nose, genitalia). Thereafter, the rest of the trunk and distal limbs	Discontinue any potential causative drug; Avoid shear force; Use topical skin antiseptics; Apply a bland, greasy emollient to the whole skin; Remove oral and nasal crusts by wet compress
Flaccid bullae	Following lesions and/or purpuric macules	Several days	Lesioned areas	Aspirate or release the blister fluid (the blister roof should be allowed to settle over the underlying dermis); Apply a bland, greasy emollient to the whole skin; Apply a nonadherent dressing to the exposed dermis
Epidermal loss	Following blistering	24 h to weeks	Blistered areas	Protect the exposed dermis and eroded mucosal surfaces; Leave detached epidermis in place to act as a biological dressing; Apply a nonadherent dressing to the exposed dermis

**Table 2:** Key points for early disease management.

Key Points
Pay close attention to pain/sedation requirements.
Only use systemic antibiotics if there is clinical evidence of infection.
To reduce epidermal detachment, use cautious handling of the patient’s skin and reduce shearing pressures.
Throughout the acute phase of TEN, collect swabs for bacterial and candidal culture from three regions of lesional skin, especially sloughy or crusted areas, on alternate days.
Avoid using blood pressure cuffs, adhesive electrocardiography leads, adhesive dressings, and identifying wrist tags to avoid epidermal harm.
Take viral swabs if herpes virus infection is suspected.

**Table 3:** Key points for symptomatic therapy.

Key Point
Early identification and withdrawal of the causative agent are mandatory.
Key point
Fluid resuscitation according to the Parkland rule is advisable during first 48 h from the onset. Further fluid input should be calculated based on diuresis, blood pressure, and invisible fluid loss.

**Table 4:** Key points for wound management.

Key Point
Nonadherent dressings should be used to avoid further epidermis destruction. Gentle manipulation of the patient, minimalization of shear forces, and irritation should be mentioned. Devitalized tissues should be gently removed avoiding injury of non-blistered areas.
Key point
Silver-containing dressings should be considered for necrotic areas or when infection of the wound bed is suspected.

## Data Availability

Not applicable.
